# Impact of broadly targeted enhanced recovery after surgery protocols in hysterectomy versus other gynecologic surgeries

**DOI:** 10.1371/journal.pone.0360744

**Published:** 2026-10-26

**Authors:** Tanya Li, Hadia Saroya, Jessica Lu, Wali U. Pirzada, Simran F. Shamith, Sina Ramtin, Asif M. Ilyas

**Affiliations:** 1 Drexel University College of Medicine, Philadelphia, Pennsylvania, United States of America; 2 Rothman Opioid Foundation, Philadelphia, Pennsylvania, United States of America; 3 Rowan-Virtua School of Osteopathic Medicine, Stratford, New Jersey, United States of America; 4 Dell Medical School at The University of Texas, Austin, Texas, United States of America; 5 Rothman Orthopaedic Institute, Philadelphia, Pennsylvania, United States of America; Cleveland Clinic, UNITED STATES OF AMERICA

## Abstract

**Introduction:**

The opioid epidemic in the United States poses a major public health challenge, with postoperative prescribing recognized as a significant source of initial opioid exposure. Midwestern states, including Iowa, have experienced substantial opioid-related morbidity and mortality, reflecting broader national trends of opioid misuse, diversion, and overdose. Excess postoperative opioid prescribing increases the availability of unused medications, which are often retained in households and pose ongoing risks for diversion and nonmedical use. These challenges highlight the need for effective, scalable strategies to reduce opioid exposure at surgical discharge. Accordingly, this study investigated the impact of a broadly targeted Enhanced Recovery After Surgery (ERAS) pathway on opioid prescribing at discharge for hysterectomy patients compared to other gynecologic procedures.

**Methods:**

A retrospective chart review was conducted across six Iowa hospitals implementing the “Billion Pill Pledge” ERAS pathway. Two groups of patients underwent surgery between November 3, 2022 and April 4, 2024: those undergoing hysterectomy (n = 63) and those undergoing other gynecologic surgeries (n = 30), including cervical hysteroscopy, cesarean section, endometriosis excision or ablation, and sterilization procedures. Pre-ERAS and post-ERAS implementation data from the postoperative period were analyzed between the two groups in terms of average opioid pill counts prescribed, percent reduction in pill counts prescribed after ERAS implementation, and percent of prescribed pills remaining unused.

**Results:**

Pre-ERAS data showed no significant difference in mean opioid pill counts prescribed at discharge between hysterectomies and other gynecologic surgeries (p = 0.3). Post-ERAS data displayed significantly higher mean pill counts for hysterectomies (23 pills) versus other gynecologic surgeries (16 pills) (p < 0.001). Patient-reported post-ERAS prescription quantities were significantly lower than surgeon-estimated pre-ERAS prescribing quantities for both hysterectomies (27 pills versus 23 pills) (p < 0.001) and other gynecologic surgeries (29 pills versus 16 pills) (p < 0.001). The hysterectomy group had a significantly smaller percent reduction in prescribed pill counts than the other gynecologic group (14% versus 40%) (p < 0.001). There was no significant difference in the proportion of prescribed pills left unused between the hysterectomy group (55%) and the other gynecologic group (65%) (p = 0.08). No prescription refills were reported within 30 days postoperatively.

**Conclusion:**

Patient-reported opioid prescription quantities prescribed at discharge following implementation of the Billion Pill Pledge ERAS pathway were significantly lower than surgeon-estimated pre-ERAS prescribing quantities across gynecologic surgeries, with a larger difference observed among non-hysterectomy procedures. Despite lower post-ERAS prescription quantities, over half of prescribed pills on average remained unused, highlighting the need for ongoing efforts to optimize postoperative opioid disposal practices. These findings suggest that broadly targeted ERAS pathways may be associated with lower postoperative opioid prescribing in gynecologic surgery.

## Introduction

Postoperative opioid prescribing in gynecologic surgery remains a key contributor to the excessive availability of narcotics, which in turn increases the risk of misuse and diversion. Studies estimate that 67% to 92% of patients have leftover opioids following surgery, with 77.5% of gynecologic and obstetric patients failing to dispose of unused pills appropriately [1]. Simultaneously, early exposure to opioids in postoperative settings has been independently associated with the development of new, persistent opioid use, particularly among younger women, a demographic often undergoing gynecologic surgeries [2]. Variability in prescribing practices, the absence of standardized guidelines, and limited patient education on opioid use and disposal highlight the need for systemic improvements in postoperative pain management, especially at discharge, where oversight is minimal [3].

In response to these challenges, standardized care models have been developed to reduce opioid exposure while maintaining effective pain control. Among these, Enhanced Recovery After Surgery (ERAS) pathways provide structured, evidence-based protocols that optimize patient outcomes by combining multimodal pain management, standardized surgical practices, and comprehensive patient education [4]. While specialty-specific ERAS pathways have shown promise in gynecologic surgery by shortening recovery times and reducing inpatient opioid needs [4], less is known about their ability to influence discharge prescribing, where many pills go unused and are at risk of diversion [5,6]. Furthermore, the majority of published ERAS pathways are customized for specific procedures or surgical specialties, limiting generalizability and demanding extensive department-level planning, development, and maintenance [7–9].

ERAS pathways have now become a global initiative, with implementation reported across North and South America, Europe, Asia, and Africa. In gynecology oncology specifically, adoption of the pathways is highest in the Americas (33%) and Europe (38%), but lower in Asia (19%) and Africa (10%) [10]. Despite these differences, ERAS pathways consistently demonstrate improved clinical outcomes, including reduced length of stay and complications [11–13]. Nonetheless, barriers such as limited resources, inconsistent adherence, and the need for local adaptation remain common worldwide [10,13].

This study evaluates the impact of a broadly implemented ERAS pathway across multiple hospitals and surgical departments, regardless of procedure or specialty. The pathway was proposed as part of the Billion Pill Pledge (BPP), an initiative to limit postoperative opioid prescriptions in the state of Iowa. This study examined the BPP ERAS pathway’s effects on postoperative opioid prescription sizes specifically in gynecologic surgery, comparing hysterectomy to other common gynecologic procedures, including cervical hysteroscopy, cesarean section, endometriosis excision or ablation, and sterilization procedures. It was hypothesized that implementation of the ERAS pathway would reduce postoperative opioid prescription sizes and the proportion of unused pills in all gynecologic surgeries. By assessing both prescription reductions and unused pill counts, the purpose of this study is to determine whether a generalized ERAS pathway can simplify implementation while still delivering meaningful reductions in opioid exposure at discharge.

## Methods

### Ethical approval

The study was approved by the Jefferson Office of Human Research Protection, with the IRB number “iRISID-2024-0830.” Approval was granted on the basis that the data analyzed in this study came from a de-identified patient database. Informed consent was waived due to the retrospective nature of the study and the use of a fully de-identified dataset provided by a third-party vendor (Goldfinch Health Inc.). The study was conducted in accordance with applicable institutional policies and ethical standards.

### Patient identification and inclusion criteria

Following institutional review board approval, a de-identified database provided by a third-party company, Goldfinch Health Inc., was retrospectively screened for patient data from hospitals in Iowa enrolled in a statewide initiative known as the Billion Pill Pledge. The Billion Pill Pledge ERAS pathway was collaboratively deployed in partnership with Goldfinch Health Inc. This retrospective review spanned patients treated across six hospitals by nine board certified gynecologic surgeons between November 2022 and April 2024, and data were accessed in May 2024. Patients included were women aged 18–80 who underwent gynecologic surgery and had complete documentation regarding postoperative opioid prescriptions and consumption. Because only patients with complete data were included, there was no attrition. Patients were categorized into hysterectomy and non-hysterectomy gynecologic surgery groups based on procedure type as recorded in the database. The non-hysterectomy group included a range of other gynecologic procedures, such as cervical D&C, cervical hysteroscopy, cesarean section, cystocele repair, endometriosis excision or ablation, oophorectomy, and sterilization procedures. The final sample size was determined by the number of eligible cases after application of exclusion criteria.

### Measuring opioid metrics

Two types of data points were analyzed per patient: a baseline (pre-ERAS) estimate and a recorded (post-ERAS) outcome. The pre-ERAS estimate represented the number of pills a patient would have been prescribed based on each surgeon’s historical practice patterns for that specific procedure prior to ERAS implementation. This estimate was extracted from the de-identified database, which was provided by the operating surgeon and reflected their typical prescribing pattern before adoption of the ERAS pathway. Because no centralized pre-ERAS prescribing dataset existed within the participating hospitals, surgeon estimates served as the sole method for approximating baseline prescribing practices. Post-ERAS data reflected actual prescriptions and consumption self-reported by patients through routine follow-up calls conducted by clinical staff, as required under new hospital order sets aligned with the ERAS pathway.

Variables extracted from the de-identified database included postoperative pills prescribed, pills consumed, and quantity of pills remaining. Due to insufficient dosage information for a subset of patients, all data were quantified in whole pill counts rather than oral morphine equivalents (OME) or morphine milligram equivalents (MME).

### Outcomes and statistical analysis

After stratification into hysterectomy and other gynecologic surgery cohorts, a percent reduction in prescription was calculated for each patient. This was the difference between pre- and post-ERAS prescribed quantity, expressed as a percentage of the pre-ERAS quantity. Proportions of prescribed opioids unused were measured for each patient as “percent of pills leftover.” Group-level averages were computed for each outcome. Additionally, two dichotomous data points were tracked: presence of a 30-day refill request and complete non-use of opioids.

Statistical comparisons of within-group changes (pre- vs. post-ERAS) used the Wilcoxon Signed-Rank test, the nonparametric alternative to the paired t-test, given the non-normal distribution of the data. Normality was assessed using the Shapiro-Wilk test, which indicated non-normal data distribution. Between-group comparisons at identical time points (e.g., post-ERAS prescriptions for hysterectomy vs. other gynecologic surgeries) used the Mann-Whitney U test. Bivariate relationships among categorical data were assessed via chi-square testing. A post hoc power analysis was performed using G*Power 3.1.9.7. Based on the observed effect size of 0.96, the study population provided 80% power (β = 0.20) at α = 0.05 to detect the observed differences between group mean values. All other analyses were performed using IBM SPSS Version 29 (IBM Corp., Armonk, N.Y.). Statistical significance was established at p < 0.05.

### Description of the ERAS pathway

Upon initiation of the Billion Pill Pledge program, each partnered hospital updated standing orders to ensure implementation of the ERAS pathway as follows:

(1) Preoperative Phase: Patients received educational materials to manage expectations around postoperative pain. Oral hydration was encouraged two hours prior to surgery using carbohydrate-rich beverages (e.g., ClearFast). Anti-inflammatory prophylaxis included meloxicam 10 mg or celecoxib 400 mg. For neuropathic pain prevention, patients were given pregabalin 75 mg or gabapentin 300 mg. Scheduled acetaminophen (1,000 mg daily for two days) was initiated preoperatively for baseline analgesia.
(2) Intraoperative Phase: Minimally invasive techniques were preferred to reduce surgical morbidity. Anesthesia protocols included neuraxial blocks and sedation, with the optional use of sugammadex for reversal of neuromuscular blockade. Liposomal bupivacaine was administered locally to provide prolonged incisional analgesia. Nausea prevention strategies included routine administration of dexamethasone, ondansetron, and/or scopolamine.
(3) Postoperative Phase: Opioid prescribing was capped at 10 doses, with non-opioid medications forming the first line of pharmacologic therapy, including acetaminophen, selective COX-2 inhibitors, gabapentinoids, and skeletal muscle relaxants (e.g., methocarbamol or cyclobenzaprine), when indicated. The 10 doses of opioids were restricted for severe breakthrough pain. Non-pharmacological interventions included routine ice application and early oral intake to promote gastrointestinal motility.

Patients also received pre-surgery kits at home that contained comfort items and education materials, including disposal tools for leftover opioids. Follow-up nurse calls reinforced pathway adherence and collected outcome data, including opioid consumption and leftover pill counts, which were entered into the de-identified database in accordance with Billion Pill Pledge standing orders.

## Results

This study included a total of 93 patients for whom procedure classification and complete pill usage data were available. Demographic information of the patient cohort was not evaluated, nor was it available as the data analyzed in this study was retrieved from a de-identified patient database provided by Goldfinch Health Inc. The hysterectomy group consisted of 63 patients, while the other gynecologic surgeries group consisted of 30 patients (Figs 1 and 2).

**Fig 1:**
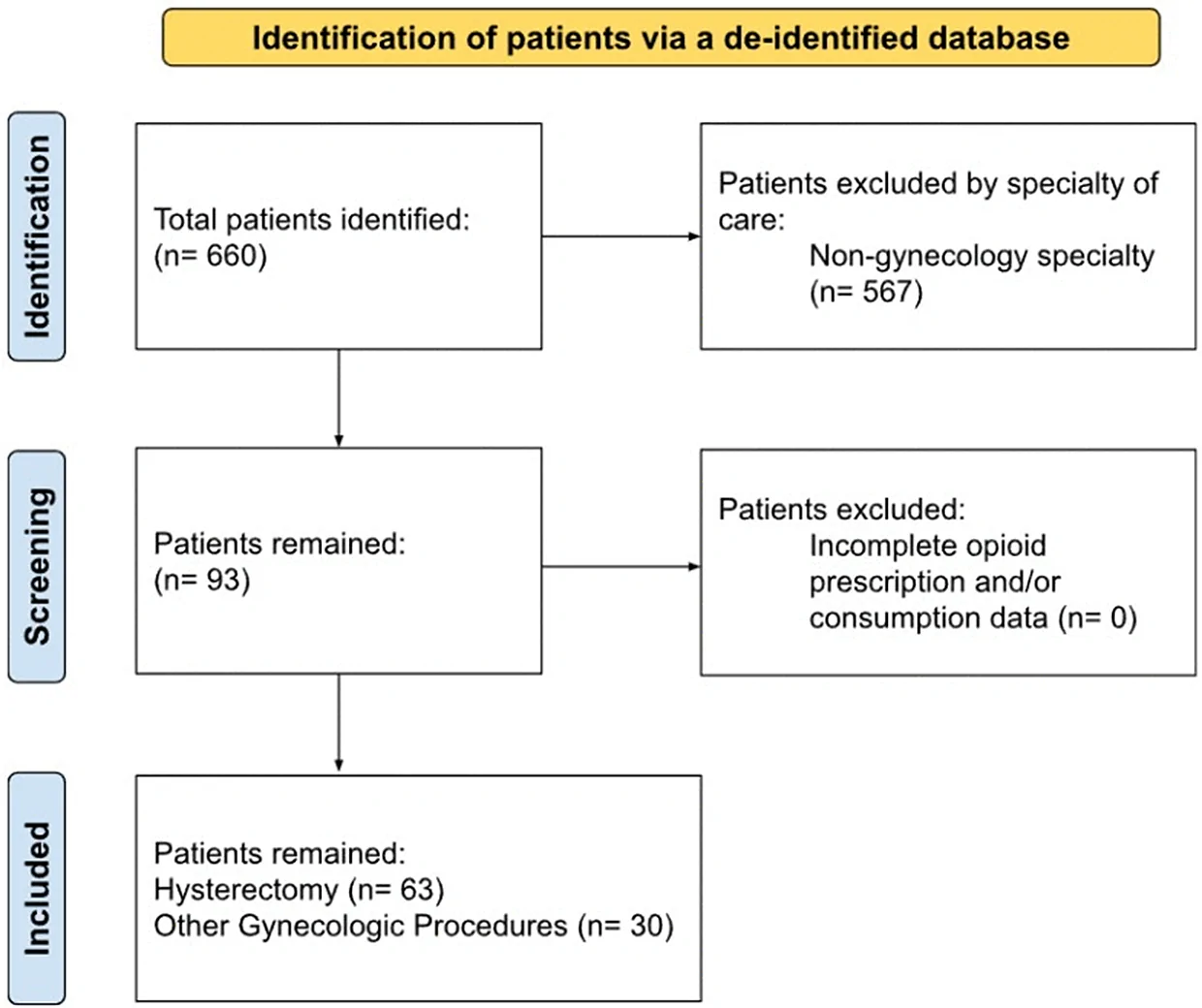
Study inclusion criteria: Flow diagram.

**Fig 2:**
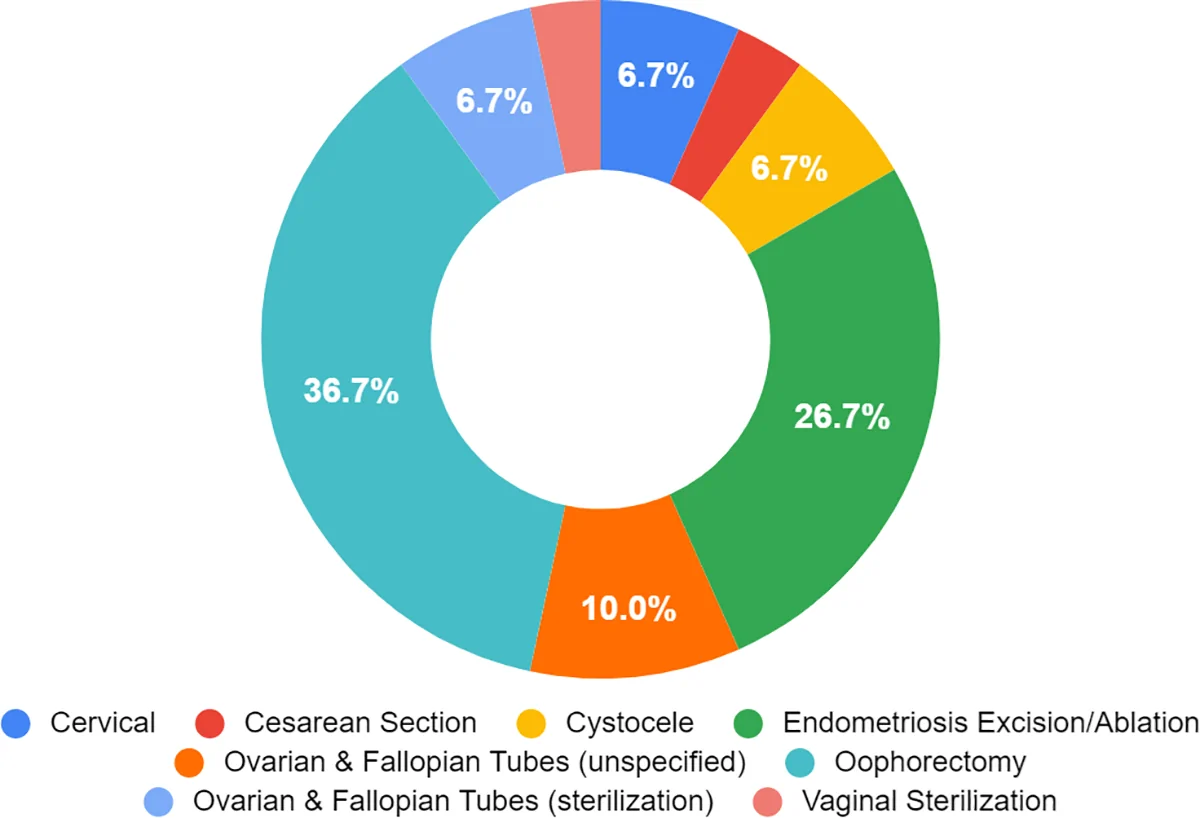
Breakdown of other procedures group: Cervical D&C (n = 1), Cervical Hysteroscopy (n = 1), Cesarean Section (n = 1), Cystocele (n = 2), Endometriosis Excision/Ablation (n = 8), Ovarian & Fallopian Tubes Procedure- Unspecified (n = 3), Oophorectomy (n = 11), Ovarian & Fallopian Tubes Sterilization (n = 2), Vaginal Sterilization (n = 1).

For the entire cohort of 93 patients, the surgeon-estimated pre-ERAS prescribing quantity was a mean of 28 pills (range: 10–40 pills). The estimated pre-ERAS mean was 27 pills (range: 10–40 pills) for hysterectomy patients, whereas the estimated mean for those in the other gynecologic surgeries group was 29 pills (range: 10–40 pills)(p = 0.3). Following ERAS implementation, the overall cohort’s patient-reported mean prescription quantity was 21 pills (range: 0–40 pills). Post-ERAS implementation, hysterectomy patients were prescribed a mean of 23 pills (range: 0–40 pills), while the other gynecologic surgeries group was prescribed a mean of 16 pills (range: 0–30 pills) (p < 0.001) (Fig 3). Patient-reported post-ERAS prescription quantities were significantly lower than surgeon-estimated pre-ERAS prescribing quantities for the entire cohort (p < 0.001). When considered individually, the hysterectomy group had an estimated pre-ERAS mean of 27 pills compared with a reported post-ERAS mean of 23 pills (p < 0.001). Similarly, the other gynecologic surgeries group had an estimated pre-ERAS mean of 29 pills compared with a reported post-ERAS mean of 16 pills (p < 0.001). These findings demonstrate lower patient-reported post-ERAS prescription quantities compared with surgeon-estimated pre-ERAS quantities, with a smaller difference observed among hysterectomy patients compared with other gynecologic surgeries (Table 1).

**Table 1:** Prescription data.

	Hysterectomy	Other Gynecologic Surgeries	p-value
No. of patients	63	30	
Mean pills prescribed pre-ERAS	27	29	0.3
Mean pills prescribed post-ERAS	23	16	<0.001
Mean % reduction from pre-ERAS	14	40	<0.001
Mean % pills leftover	55	65	0.08

Patient groups with corresponding mean pre-ERAS and post-ERAS opioid prescription quantity, as well as mean percent reduction in prescriptions.

**Fig 3:**
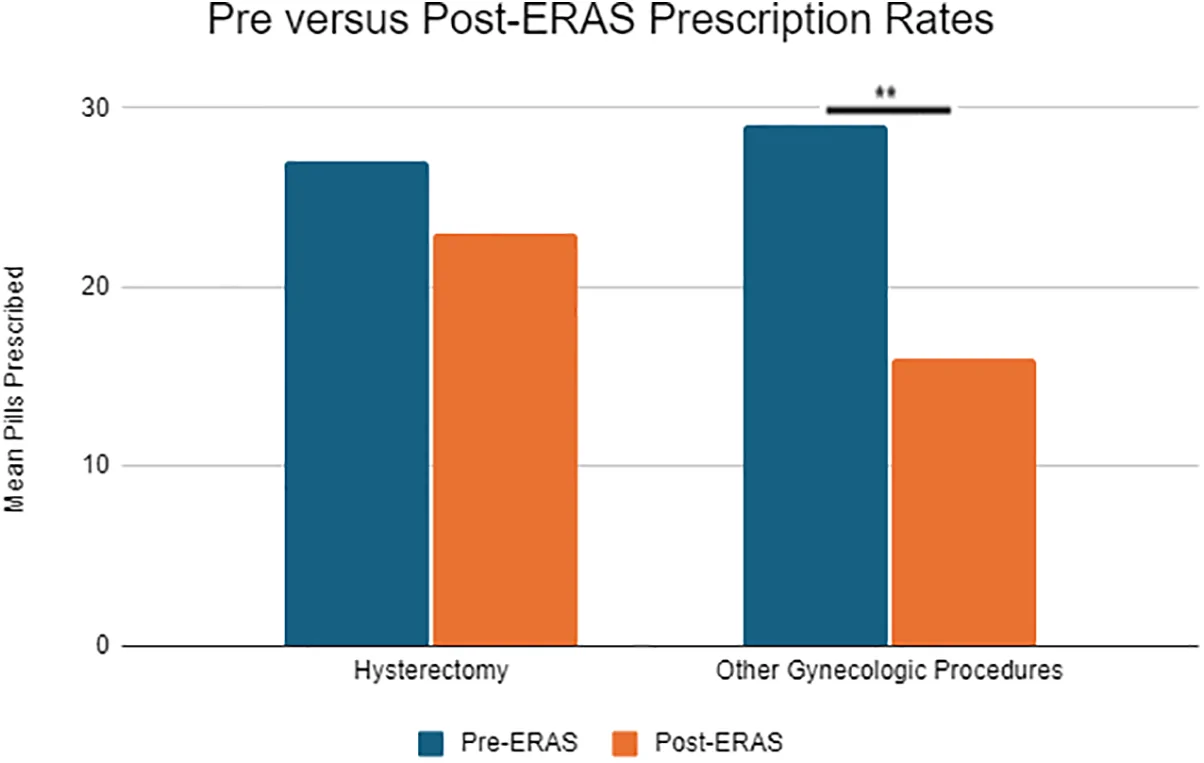
Bar graph comparing mean pre-ERAS and post-ERAS opioid prescription rates by surgical procedure.

The overall cohort displayed a mean percent reduction in prescription size of 23% between surgeon-estimated pre-ERAS and patient-reported post-ERAS prescription quantities. The hysterectomy group showed a mean reduction of 14%, while the other gynecologic surgeries group exhibited a mean reduction of 40%. Upon statistical analysis, the two groups demonstrated significantly different mean percent reductions, with a greater difference observed among non-hysterectomy procedures compared with hysterectomies (p < 0.001).

The mean quantity of pills consumed was significantly higher in patients undergoing hysterectomy compared to those undergoing other surgeries. Hysterectomy patients consumed a mean of 9 pills (range: 0–40 pills), while the other surgeries group consumed a mean of 5 pills (range: 0–20 pills), reflecting higher postoperative analgesic needs for patients undergoing hysterectomies (p < 0.003). Additionally, the other surgeries group reported a mean of 11 pills remaining (range: 0–28 pills), compared to 13 pills remaining for hysterectomy patients (range: 0–39 pills) (p < 0.3). Hysterectomy patients presented with a mean percent pills leftover of 55%, compared to a mean percent pills leftover of 65% for the other surgeries group (p < 0.08), indicating that a substantial portion of prescribed opioids remained unused in both groups. This difference did not reach statistical significance at the p < 0.05 level (Table 2).

**Table 2:** Opioid consumption data.

	Hysterectomy	Other Gynecologic Surgeries	p-value
No. of patients	63	30	
Mean quantity utilized	9	5	0.003
Mean remaining quantity	13	11	0.3
Mean % pills leftover	55	65	0.08

Patients sorted by specialty of care with corresponding mean opioids consumed, mean opioids unused, and mean % of prescribed pills leftover.

Eight out of 63 patients in the hysterectomy group reported consuming zero opioids from their postoperatively prescribed amount (Fig 4). Comparatively, eight of 30 patients in the other gynecologic surgeries group reported zero opioids consumed. The chi-square statistic for this comparison was 2.8, with a corresponding p-value of 0.1. Although these findings did not reach statistical significance at the p < 0.05 level, it suggests that a proportion of patients across procedures did not require any opioids postoperatively. Importantly, patient records across the cohort showed no incidence of a refill within the 30-day postoperative period, indicating that initial prescriptions were generally sufficient and that postoperative opioid exposure did not extend into chronic use.

**Fig 4:**
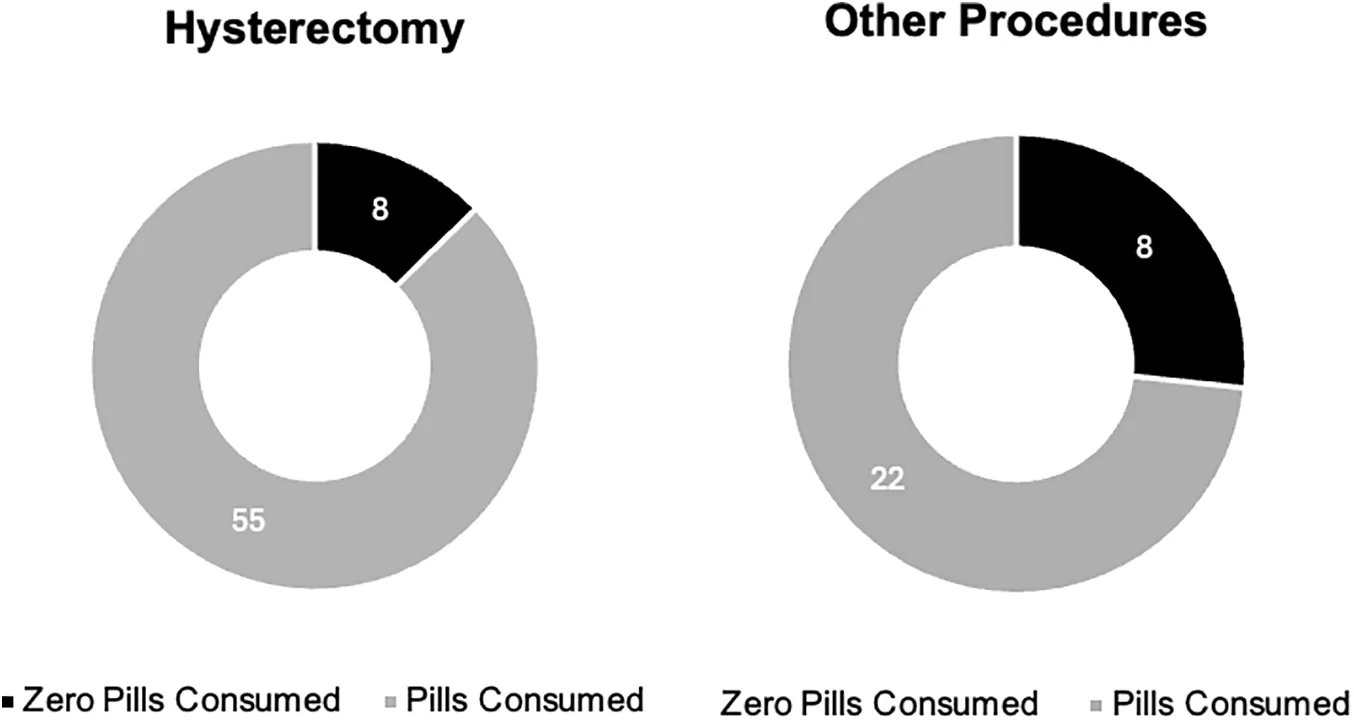
Incidence of zero pills consumed.

## Discussion

Enhanced Recovery After Surgery (ERAS) pathways have consistently demonstrated benefits in gynecologic surgery, including improved pain scores and shorter hospital stays [14,15]. In hysterectomies and minimally invasive gynecologic oncology procedures specifically, ERAS pathways have been associated with decreased intraoperative and postoperative opioid use while maintaining or improving pain management [16,17]. Despite these advances, relatively few studies have examined post-discharge prescribing patterns, patient consumption behaviors, or leftover medication quantities, which are factors critical to addressing long-term opioid use and diversion risks. This gap is particularly relevant for younger women undergoing gynecologic surgeries, who are vulnerable to persistent opioid use and dependence [18,19].

Most prior studies focus narrowly on hysterectomies, with limited evaluation of ERAS implementation across other gynecologic procedures [20–23]. Consequently, little is known about how ERAS pathways influence opioid prescribing on a multi-procedural scale or how prescription sizes compare with actual patient use. This study therefore assesses ERAS implementation across a variety of gynecologic surgeries, including less frequently studied procedures such as ovarian and adnexal surgeries, and examines patient-centered outcomes such as unused pill quantities and percent reductions rather than oral morphine equivalents (OME) or morphine milligram equivalents (MME) alone.

In this study, postoperative opioid prescription quantities were lower following ERAS implementation across gynecologic surgeries, with a larger difference between estimated pre-ERAS and reported post-ERAS quantities among non-hysterectomy procedures. Higher opioid use after hysterectomies may reflect greater surgical complexity, longer operative times, and higher complication rates, such as urinary tract injuries and blood loss that requires transfusions [24]. Since prolonged hysterectomies are also linked to increased infections, thrombosis, and readmissions [25], multiple factors likely contribute to greater postoperative pain and analgesic needs compared with other minimally invasive gynecologic surgeries.

Overprescription of opioids is also well-documented across surgical specialties [26–28], and current evidence suggests this pattern extends to gynecologic procedures. Studies of pelvic organ prolapse and other urogynecologic procedures show that patients often receive significantly more opioids than they consume [29,30]. The larger difference between surgeon-estimated pre-ERAS and patient-reported post-ERAS prescription quantities for non-hysterectomy surgeries may reflect greater baseline prescribing relative to postoperative opioid needs. Because the non-hysterectomy cohort included procedures with varying pain profiles and analgesic requirements, the observed 40% reduction may reflect differences in baseline prescribing across procedures and should not be interpreted as uniform across all procedures within this group. Since the pre-ERAS quantities were retrospectively estimated, the difference between the estimated pre-ERAS and reported post-ERAS prescription quantities also should not be interpreted as a directly observed response to ERAS implementation. In contrast, the smaller difference in prescription quantities observed following ERAS pathway implementation post-hysterectomy may be due to true opioid use more closely matching prescription sizes.

Importantly, lower postoperative opioid consumption and prescribing should be interpreted in the context of adequate postoperative pain control. Reduction in opioid prescribing represents a meaningful clinical benefit only if pain control, patient satisfaction, and functional recovery are maintained or improved. In this study, pain scores, patient satisfaction, functional recovery, and complication rates were not available, so it cannot be determined whether lower post-ERAS prescribing was accompanied by maintained or improved pain control, particularly after hysterectomies, where analgesic requirement may be greater. Future studies incorporating patient-centered pain and recovery outcomes are needed to more fully evaluate the clinical benefit of ERAS-based opioid prescribing practices.

Most ERAS research within the field of gynecologic surgery has focused almost exclusively on hysterectomies, while no major gynecologic society has established opioid prescribing guidelines for minimally invasive surgeries [29]. Therefore, understanding of how multimodal pathways influence prescribing and pain management across the broader spectrum of gynecologic procedures remains limited. Further research is also warranted to evaluate procedure-specific responses to ERAS-based prescriptions and to establish guidelines that better align with patient needs.

Prior literature on pre-ERAS postoperative opioid prescriptions shows wide variability for minimally invasive hysterectomies, with ranges from 35 to 161 OME [17,31,32]. Data for other gynecologic surgeries, while sparse, have been reported to fall within similar OME ranges [20]. Due to a lack of dosage information, this current study reported opioid data only in absolute pills. Though pill counts more accurately reflect units at risk for drug diversion, this prevents any direct comparisons with prior studies that report opioid measures in OME or MME. In this study, further stratification of the other gynecologic surgeries group by procedure type was not feasible due to insufficient sample sizes and incomplete opioid prescription and consumption data.

As seen in this study, patient-reported postoperative opioid prescription quantities were significantly lower than surgeon-estimated pre-ERAS prescribing quantities across both hysterectomy and other gynecologic surgery groups, suggesting the potential broad applicability of a single set of ERAS pathways. Observed percent reductions align broadly with prior literature reporting opioid reductions of 30%−70% following multimodal interventions [2,17,33–35]. However, variability in study design, outcome measures, protocol specifics, and the retrospective estimation of the pre-ERAS baseline in this study complicate direct comparisons. The variation in mean percent reduction across procedure types in this current study suggests that opioid prescribing within ERAS pathways may benefit from being tailored to the specific surgical procedure. Nonetheless, the results of this study suggest that the blanket implementation of a broadly targeted ERAS pathway may provide a useful starting point for promoting lower postoperative opioid prescription quantities across various procedure types within a surgical field.

Despite reductions in prescription sizes, more than half of prescribed opioids remained unused in both cohorts, reflecting ongoing excess post-ERAS implementation. This is consistent with prior ERAS studies in gynecology, where 50%−75% of prescribed opioids are typically unused [36,37]. Due to a lack of patient reported consumption rates at the pre-ERAS timepoint, this study was not able to capture a baseline in order to gauge the effectiveness of the ERAS pathway at reducing percent of opioids leftover. However, non-ERAS studies have reported leftover rates similar to those reported by this study’s post-ERAS data [38,39]. When contextualized, these findings underscore that ERAS pathways do not fully eliminate overprescribing, highlighting the persistent challenge of leftover opioids and the risk of diversion.

This study has several limitations. Pre-ERAS prescription data were based on surgeon estimates rather than patient-reported data because the de-identified database was only created after ERAS implementation at partner hospital sites, which may introduce recall bias and provider variability. Post-ERAS data, in contrast, were based on patient self-report collected through structured follow-up calls, resulting in methodologically non-equivalent data sources. No validation or sensitivity analysis was available to determine how closely surgeon-estimated pre-ERAS prescribing approximated actual historical prescribing patterns. Therefore, the magnitude of the observed pre-post differences should be interpreted with caution. A patient-reported pre-ERAS dataset would have allowed for a more robust comparison between control and experimental groups. Postoperative clinical outcomes such as pain scores, patient satisfaction, and functional recovery were also not available; therefore, patient response was evaluated solely through opioid consumption and unused pill counts. Furthermore, all 93 patients in this study received the ERAS intervention, and there was no concurrent standard-care comparison group. As a result, the observed differences cannot be attributed solely to the BPP ERAS pathway and may have been influenced by broader trends in opioid prescribing during the 2022–2024 study period, including evolving prescribing practices, institutional policy changes, and regulatory efforts to reduce opioid use. Because these secular trends could not be separated from the effects of ERAS implementation, they represent a potential confounding factor in the interpretation of the pre-post comparison. The use of a post hoc power analysis is another limitation, as the effect size was derived from the same data being analyzed. Therefore, while the analysis indicates that the observed effect was detectable within the available sample, it does not prospectively justify the sample size or study design.

Additionally, opioid consumption data were only available post-ERAS, limiting assessment of changes in leftover pill quantities. Although these quantities were recorded post-ERAS, the study did not capture whether patients properly disposed of unused opioids or their disposal methods. Future studies could include patient-reported disposal behaviors to better assess risk reduction strategies and inform interventions such as patient counseling and medication take-back programs. The absence of patient-level demographic and clinical variables also precluded adjustment for potential confounders affecting prescribing and consumption patterns. Furthermore, because dosage information and timing of opioid use were not available, whole pill counts were analyzed rather than OME or MME, while postoperative refill rates served as a proxy for prolonged opioid exposure. Future studies should report both metrics to account for opioid type, dosage, duration, leftover quantities, and potential diversion risk. Finally, stratification by individual gynecologic procedures was not possible due to insufficient numbers within each subgroup, so procedures were grouped into hysterectomy and other gynecologic surgery categories only. Therefore, the observed 40% reduction between surgeon-estimated pre-ERAS and patient-reported post-ERAS prescription quantities represents an aggregate finding and should not be generalized uniformly across individual procedures. Future studies with larger sample sizes should consider procedure-specific analyses to evaluate differential responses to ERAS implementation.

## Conclusion

This study suggests that a broadly-implemented ERAS pathway may be associated with lower postoperative opioid prescription quantities across gynecologic surgeries. However, the difference between pre-ERAS and post-ERAS prescribing quantities was less pronounced in hysterectomy patients relative to other gynecologic surgeries, which may reflect higher pain control needs, surgical complexity, or prescriptions already closely matching actual use. By including a broader range of gynecologic surgeries and assessing pill counts and leftover opioids, this study adds to the existing literature that has largely focused on hysterectomies alone. Despite lower post-ERAS prescribing quantities, the persistence of high rates of unused opioids emphasizes ongoing risks for diversion and suggests that ERAS pathways alone may not fully resolve overprescribing. These findings underscore the need for procedure-specific prescribing strategies and targeted opioid stewardship to better align prescriptions with patient needs.
